# A Novel Biosurfactant Produced by *Aureobasidium pullulans* L3-GPY from a Tiger Lily Wild Flower, *Lilium lancifolium* Thunb.

**DOI:** 10.1371/journal.pone.0122917

**Published:** 2015-04-07

**Authors:** Jong Shik Kim, In Kyoung Lee, Bong Sik Yun

**Affiliations:** 1 Gyeongbuk Institute for Marine Bioindustry, 22 Haeyanggwahak-gil, Uljin, Gyeongbuk 767–813, Republic of Korea; 2 Division of Biotechnology and Advanced Institute of Environmental and Bioscience, Chonbuk National University, 79 Gobong-ro, Iksan, Chonbuk 570–752, Republic of Korea; University of Huddersfield, UNITED KINGDOM

## Abstract

Yeast biosurfactants are important biotechnological products in the food industry, and they have medical and cosmeceutical applications owing to their specific modes of action, low toxicity, and applicability. Thus, we have isolated and examined biosurfactant-producing yeast for various industrial and medical applications. A rapid and simple method was developed to screen biosurfactant-producing yeasts for high production of eco-friendly biosurfactants. Using this method, several potential niches of biosurfactant-producing yeasts, such as wild flowers, were investigated. We successfully selected a yeast strain, L3-GPY, with potent surfactant activity from a tiger lily, *Lilium lancifolium* Thunb. Here, we report the first identification of strain L3-GPY as the black yeast *Aureobasidium pullulans*. In addition, we isolated a new low-surface-tension chemical, designated glycerol-liamocin, from the culture supernatant of strain L3-GPY through consecutive chromatography steps, involving an ODS column, solvent partition, silica gel, Sephadex LH-20, and an ODS Sep-Pak cartridge column. The chemical structure of glycerol-liamocin, determined by mass spectrometry and nuclear magnetic resonance spectroscopy, indicates that it is a novel compound with the molecular formula C_33_H_62_O_12_. Furthermore, glycerol-liamocin exhibited potent biosurfactant activity (31 mN/m). These results suggest that glycerol-liamocin is a potential novel biosurfactantfor use in various industrial applications.

## Introduction

A surfactant molecule has both hydrophilic and hydrophobic moieties that can accumulate at interfaces, reduce surface and interfacial tensions, and form aggregate structures such as micelles, which consist of external hydrophilic moieties and internal hydrophobic moieties [1–6]. In recent years, increasing concern has been placed on the environmental impacts of chemical surfactants [6]. Biosurfactants produced intracellularly or extracellularly by microorganisms such as yeast, fungi, and bacteria are biodegradable and eco-friendly materials having lower toxicity than synthetic surfactants [1–6], and biosurfactants have been developed as suitable alternatives for antimicrobial and biomedical agents [7]. Biosurfactants produced by yeasts have many potential applications, because products with a “generally regarded as safe” (GRAS) status are not toxic or pathogenic, allowing for use in food and pharmaceutical industries [8]. Furthermore, biosurfactants are used for specific purposes, depending on their complex chemical structures, and they are useful in the same applications as synthetic surfactants, with similar physical and chemical properties such as reducing surface tension and improving stability at various temperatures and pH [1–6]. Therefore, biosurfactants have been widely used in various industrial fields including the fields of medicine, food, cosmetics, detergent, pulp and paper, secondary recovery of crude oil, onshore and offshore oil decontamination, and oil and fat degradation [4, 5].

During the screening for biosurfactant-producing yeasts, we isolated a yeast strain, which was identified as *Aureobasidium pullulans*, from a *Lilium lancifolium* Thunb. tiger lily. *A*. *pullulans* is ubiquitous yeast reportedly found in various locations, including soil, water, plant leaves, painted walls, bathroom surfaces, hypersaline water in solar salterns, and Arctic glacier ice [8–11]. *A*. *pullulans* produces polysaccharides such as pullulan and β-glucan, which have been utilized previously for various industrial and medical applications [12–15]. Recently, there have been reports on *A*. *pullulans* with regard to (poly) malic acid [16–22], lipase [23], laccase [24], mannitol oils [25], biocontrol [26, 27], lipid composition [28, 29], and siderophores [30]. *A*. *pullulans* strains also reportedly produce biosurfactants of heavy oils [31]. The *A*. *pullulans* L3-GPY strain that we isolated exhibited potent biosurfactant activity. In this report, we describe the phylogenetic identification of this biosurfactant-producing yeast, isolation and structural determination of the active compound (glycerol-liamocin), and the biosurfactant activity.

## Materials and Methods

### Screening of potential biosurfactant-producing yeast

The tiger lily is perennial plant species that flowers each summer and is widely distributed in residential areas and non-residential areas in Korea. We sampled tiger lilies without specific permission, because no specific permissions for sample collection were required under local and national laws. Sampling site is located at N 36° 59' 54.49" and E 129° 24' 17.28".

Media for yeast-screening included the following types: DG18 agar (Dichloran-Glycerol 18%; MB Cell, Seoul), DOB (Drop Out Base) with CSM (Complete Amino Acid Supplement Mixture) agar (MP Bio, CA, USA), GPY agar (4% glucose, 0.5% peptone, 0.5% yeast extract, and 1.5% agar), and SCG agar (Sabouraud Chloramphenicol Gentamicin; MB Cell, Seoul). Antibiotics (100 mg/L chloramphenicol and streptomycin) were added to each medium to repress bacterial growth, and 0.1% Triton X-100 and 0.4% l-sorbose were added to repress fungal growth. Yeasts were cultured on DG18, DOB with CSM, GPY, and SCG agar media in square plates (245 × 245 × 25 mm, Nunc Bio-Assay Dish; Thermo Scientific, Roskilde, Denmark).

*L*. *lancifolium* (formerly *L*. *tigrinum*) samples were aseptically collected from a residential area in Uljin by using autoclaved scissors and forceps and were placed in clean plastic bags. The samples were stored in a cooler during transfer to the laboratory in Uljin and then processed the same day. Each sample was washed 3 times with 0.1 M potassium phosphate buffer and stored in an autoclaved container. Several washed leaf samples were placed in tubes (Falcon, Los Angeles, CA) with a maximum volume of 10 mL of 10 mM potassium phosphate buffer, and samples were homogenized using an autoclavable hand homogenizer (T10 basis; IKA, Germany). Homogenized samples (1 mL) were placed on sterile solid media, plated using a glass spreader, and incubated at 25°C for 2–5 d. Yeast colonies that grew were selected with an autoclaved toothpick and inoculated into 96-deep-well plates for liquid culture at 25°C and 800 rpm for 48 h. Drops of culture broth (20 μL) were tested with the drop-collapse method on hydrophobic parafilm using an 8-channel pipette, and the size of the droplet was confirmed by comparison with water; the spreading diameters of droplets on parafilm were measured upon addition of culture fluid. Selected biosurfactant producers were streaked three times on agar plates to isolate pure yeast colonies, and the isolates were cultured repeatedly in GPY liquid medium and on agar plates with 20% glucose until single, separate colonies grew, to avoid cross-contamination during cultivation. The yeast selected for further studies was deposited in the Korean Culture Center of Microorganisms (KCCM) and cultured on GPY with glucose as a carbon source.

### Sequencing of the yeast isolate

The primer sets used to amplify 18S rRNA and elongase (*ELO*) genes for the *A*. *pullulans* L3-GPY strain are described in previous reports: NS1, NS3, and NS8 [32] and ELO2-F and ELO2-R [33], respectively. For PCR, DNA was extracted from an *A*. *pullulans* L3-GPY colony growing on an agar plate by the Instagene^TM^ Matrix method, according to the manufacturer’s protocol (Bio-Rad Laboratories, Hercules, CA).

PCR was performed with 20 ng of genomic DNA as the template in a 30-μL reaction mixture with EF-*Taq* DNA polymerase (Solgent, Korea). The PCR program included the following steps: 95°C for 5 min; followed by 35 cycles of 95°C for 2 min, 55°C for 60 s, and 72°C for 60 s; and a final extension step for 10 min at 72°C. Thereafter, the amplification products were purified using a multiscreen filter plate (Millipore Corp., Bedford, MA). Sequencing reactions were performed using a PRISM BigDye Terminator v3.1 Cycle Sequencing Kit (Applied Biosystems, Foster City, CA). Hi-Di formamide (Applied Biosystems) was added to the DNA samples containing the extension products. The mixture was incubated at 95°C for 5 min, followed by 5 min on ice, and then analyzed using an ABI Prism 3730XL DNA Analyzer (Applied Biosystems). DNA sequencing of isolates was performed by Macrogen Inc. (Seoul, Korea). The nucleotide sequences reported in this paper were deposited in DDBJ/GenBank under the following accession numbers: AB746215 (18S rRNA) and AB746250 (*ELO*).

### Phylogenetic analysis of *A*. *pullulans* isolate L3-GPY

Nucleotide sequences of 18S rRNA and *ELO* genes were aligned using the ClustalW2 program on the EMBL-EBI website. A BLAST search was used to identify GenBank sequences representing the most closely related strain to *A*. *pullulans* L3-GPY. Phylogenetic trees were constructed by the neighbor-joining method [34] using MEGA5 for Windows, including bootstrap analyses based on 1000 samples, and evolutionary distances were calculated with the Kimura 2-parameter method [35].

### Separation and purification of glycerol-liamocin from *A*. *pullulans* L3-GPY

**Fig 1:**
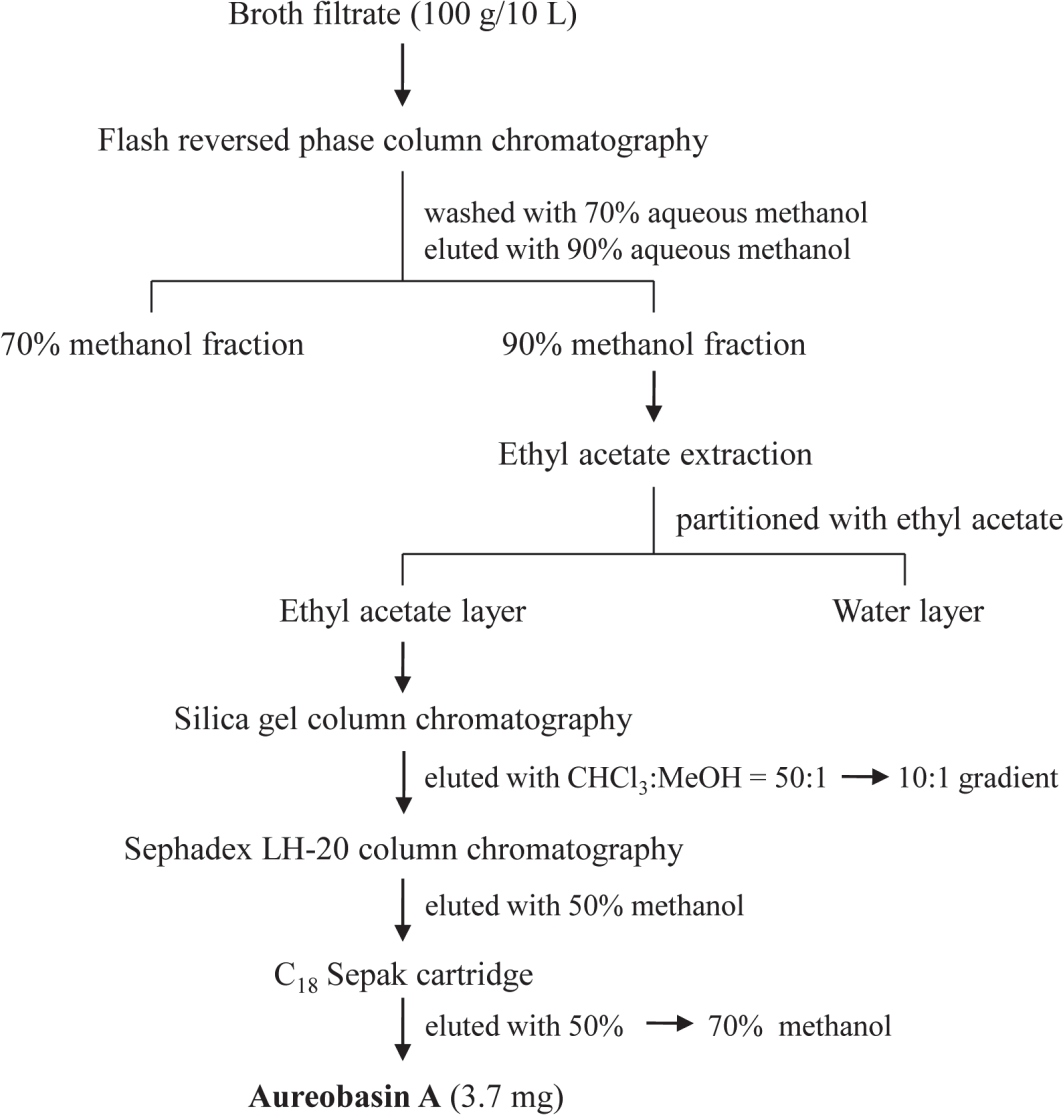
Procedure for isolation and purification of glycerol-liamocin.

Culture broth from *A*. *pullulans* L3-GPY was lyophilized, dissolved in water, and filtered with gauze. The filtrate was subjected to flash C18 reversed-phase (**Phenomenex**, **Torrance**, **CA**, USA) column chromatography. The column was washed with 70% aqueous methanol and then eluted with 90% aqueous methanol. The eluate was concentrated under reduced pressure to remove methanol, and the aqueous concentrate was partitioned with ethyl acetate. Surfactant activity was detected in the ethyl acetate-soluble portion. Then, the ethyl acetate-soluble phase was concentrated under reduced pressure, and the concentrate was separated on a column of silica gel (**Sigma-Aldrich**, St. Louis, MO, USA) and eluted with an increasing amount of methanol in chloroform (CHCl_3_:MeOH, 50:1 to 10:1 [v/v]). Fractions showing activity were combined, concentrated, subjected to Sephadex LH-20 (Amersham Biosciences, Uppsala, Sweden) column chromatography, and eluted with 50% aqueous methanol. After the activity of fractions was evaluated, active fractions were collected and concentrated under reduced pressure. Then, the concentrate was separated on a Sep-Pak C_18_ cartridge (Waters, Milford, MA) and eluted with a gradient from 50%–70% aqueous methanol to yield the purified, active compound, glycerol-liamocin (3.7 mg). The isolation and purification procedures for glycerol-liamocin are depicted in Fig 1.

### Structure analysis of glycerol-liamocin

To determine the chemical structure of glycerol-liamocin, the high-resolution mass was determined using electrospray ionization (ESI)-QTRAP-3200 Mass Spectrometer (ESI-MS; Applied Biosystems). Ultraviolet (UV) and infrared (IR) spectra were recorded on Shimadzu UV-300 and FT-IR Equinox 55 spectrophotometers, respectively. Nuclear magnetic resonance (NMR) spectra were obtained on a JEOL JNM-ECA600, 600 MHz FT-NMR Spectrometer at 600 MHz for ^1^H NMR and at 150 MHz for ^13^C NMR in CD_3_OD. Chemical shifts are given in ppm (*δ*), with tetramethylsilane as the internal standard.

### Surfactant activity of glycerol-liamocin

Surfactant activity was determined by dissolving the compound in water, loading 20 μL of the resulting solution on a hydrophobic support (parafilm), and measuring the degree of spreading as increasing droplet diameter size. Increased spreading was considered to represent increased biosurfactant activity, because of decreased surface tension [36–38]. An equal amount of distilled water was used as a control. Surface tension (N/m) variation was measured using a Sigma Model 700 Tensiometer (KSV Instruments Ltd., Helsinki, Finland).

## Results and Discussion

### Selection and phylogenetic identification of a novel biosurfactant producer

**Fig 2:**
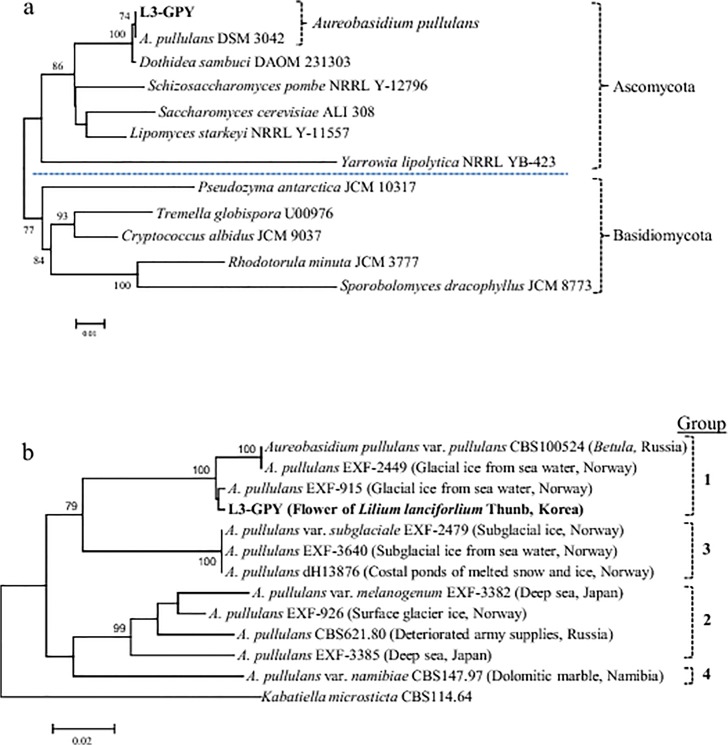
Phylogenetic trees of (a) 18S rRNA gene and (b) elongase gene (*ELO*) from *A*. *pullulans* L3-GPY with reference sequences. Bootstrap cutoff values of 75% are noted at the branch junctions.

We isolated a yeast strain, L3-GPY, with potent surface activity from a tiger lily, *Lilium lancifolium* Thunb, which was screened among other yeast isolates from the homogenized flower samples by the drop-collapse test. Our phylogenetic analysis of 18S rRNA gene sequences from genomic DNA of biosurfactant producer L3-GPY showed that this isolate is an ascomycetous yeast belonging to class Dothideomycetes, order Dothideales, and family Dothioraceae. The black yeast is the closest species to *A*. *pullulans*. Additionally, *A*. *pullulans* gene sequences share high similarity with many 18S rRNA genes of yeast and fungi. Therefore, the yeast could not be identified based on only the 18S rRNA genes (Fig 2A).

To identify to which species or interspecies the strain belongs, a deep clade phylogenetic analysis of L3-GPY*ELO* was performed, which revealed that the yeast strain is most closely related to Group 1 of *A*. *pullulans* interspecies, together with *A*. *pullulans* var. *pullulans* (Fig 2B).

The NCBI BLAST search results showed 98% or 99% identity with the group, including *A*. *pullulans* EXF-915 and EXF-2449, and the results showed 91% identity with *A*. *pullulans* var. *subglaciale*, which is in Group 3, including EXF-2479 and EXF-3640. The strain has ≤90% identity with *A*. *pullulans* var. *melanogenum*, which is in Group 2. Both the BLAST search and phylogenetic analyses of 18S rRNA and *ELO* genes suggested that the biosurfactant producer should be classified in Group 1, which includes *A*. *pullulans* var. *pullulans*, based on results of the previous study by Zalar et al. [33]. To date, studies have focused on *A*. *pullulans* production of pullulan and other chemicals; hence, this report reveals that *A*. *pullulans* also produces a novel type biosurfactant.

### Chemical structure of glycerol-liamocin

**Fig 3:**
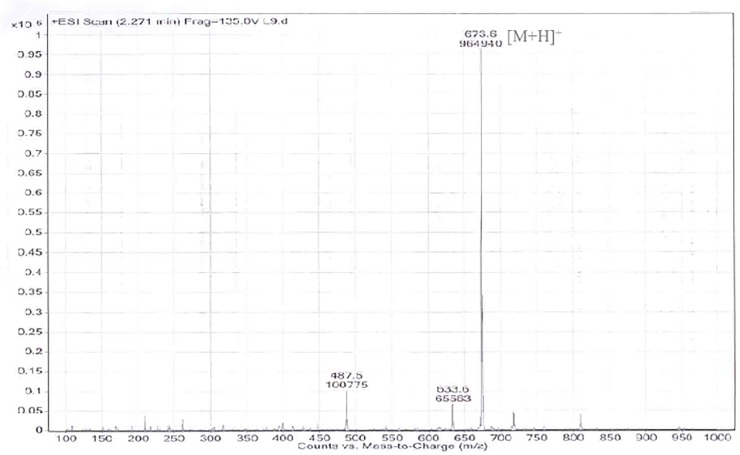
ESI-mass spectrum of glycerol-liamocin from *A*. *pullulans* L3-GPY.

The chemical structure, as determined by MS and NMR data, of the active compound glycerol-liamocin, represents a novel biosurfactant. Glycerol-liamocin was obtained as a colorless glycolipid. The molecular weight of glycerol-liamocin was determined to be 650 g/mol by ESI-MS measurement, which provided a quasi-molecular ion peak at mass: charge (*m/z*) 673.6 [M+Na]^+^ in positive mode (Fig 3). The molecular formula was established to be C_33_H_62_O_12_ by high-resolution ESI-MS measurement (*m/z* 673.4160 [M+Na]^+^, *Δ*+2.1 mmu) in combination with ^1^H and ^13^C NMR data. This molecular formula dictates 3 degrees of unsaturation.

The ^1^H NMR spectrum of glycerol-liamocin measured in CD_3_OD shows signals from seven oxygenated methines and two oxygenated methylenes at δ 3.5–5.1, three methylenes at δ 2.4–2.6, 15 methylenes from alkyl chains at δ 1.3–1.8, and three methyls at δ 0.91 (Table 1).

**Table 1:** ^1^H and ^13^C NMR spectral data of glycerol-liamocin in CD_3_OD^*a*^.

no.	δ_C_	δ_H_
1	171.8	
2	41.8	2.53(m), 2.42(m)^*b*^
3	65.5	4.19(m)
4	40.9	1.81(m), 1.75(m)
5	71.9	5.07(m)
6	33.8	1.62(m)
7	24.6	1.3–1.5(m)
8	31.5	1.3–1.5(m)
9	22.4	1.3–1.5(m)
10	13.1	0.91
1′	171.8	
2′	42.2	2.53(m), 2.42(m)
3′	65.4	4.09(m)
4′	40.9	1.81(m), 1.75(m)
5′	71.9	5.07(m)
6′	33.8	1.62 (m)
7′	24.6	1.3–1.5(m)
8′	31.5	1.3–1.5(m)
9′	22.4	1.3–1.5(m)
10′	13.1	0.91
1″	171.8	
2″	42.3	2.53(m), 2.42(m)
3″	67.1	4.19(m)
4″	43.3	1.62(m)
5″	69.7	3.74(m)
6″	37.2	1.45(m)
7″	24.9	1.3–1.5(m)
8″	31.8	1.3–1.5(m)
9″	22.3	1.3–1.5(m)
10″	13.1	0.91
Gly1	62.7	3.56(m)
2	69.7	3.83(m)
3	65.3	4.19, 4.09(m)

^*a*^NMR data were recorded at 600 MHz for proton and at 150 MHz for carbon.

^*b*^Proton resonance multiplicity in parentheses.

**Fig 4:**
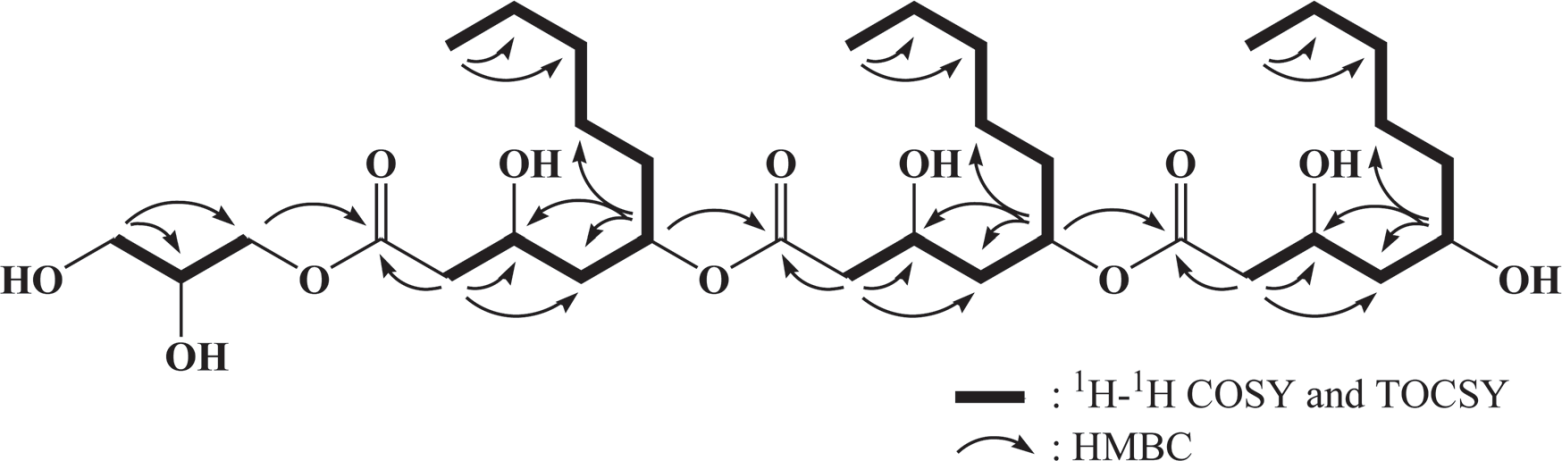
Chemical structure of glycerol-liamocin elucidated by two-dimensional NMR experiments.

Although the ^13^C NMR spectrum displays many overlapping signals, 33 carbon signals were assigned based on DEPT and HMQC spectra. All proton-bearing carbons were verified by the HMQC spectrum, and the ^13^C NMR spectrum in combination with DEPT and HMQC spectra displays three overlapping ester carbonyl carbons at δ 171.8; seven oxygenated methine carbons at δ 71.9 (×2), 69.7 (×2), 67.1, 65.5, and 65.4; two oxygenated methylene carbons at δ 65.3 and 62.7; 18 methylene carbons at δ 22–43; and three methyl carbons at δ 13.1. The ^1^H-^1^H COSY and TOCSY spectra established three similar partial structures, two 3,5-dihydroxydecanoyl moieties and one glycerol (Fig 4). The C-5 positions of two 3,5-dihydroxydecanoyl groups should be acylated, based on the proton chemical shift values appearing downfield of δ_H_ 5.07. The overall chemical structure of glycerol-liamocin was determined by the HMBC spectrum (Fig 4). Long-range correlations of the methylene protons at δ 4.19 and 4.09 and the methylene protons at δ 2.53 and 2.42 to the carbonyl carbon at δ 171.8 (C-1) revealed that the glycerol moiety is attached to C-1 via an ester linkage. The methine proton at δ 5.07 and the methylene protons at δ 2.53 and 2.42 exhibited long-range correlations to the carbonyl carbon at δ 171.8 (C-2), suggesting that three 3,5-dihydroxydecanoyl moieties are connected to each other through ester linkages between the 1- and 5-positons. As a result of our literature survey and structural investigations, we determined that glycerol-liamocin a new glycerol derivative of 3,5-dihydroxydecanoic polyesters, such as exophilin A and liamocins, which were previously isolated as antibacterial compounds from the culture broth of *Exophiala pisciphila* NI10102 [39] and novel extracellular mannitol oils from *A*. *pullulans* NRRL50380 [25], respectively. The NMR spectral data of 3,5-dihydroxydecanoyl moieties are well matched with those of exophilin A and liamocins [25, 39].

### Surface tension properties of glycerol-liamocin

**Fig 5:**
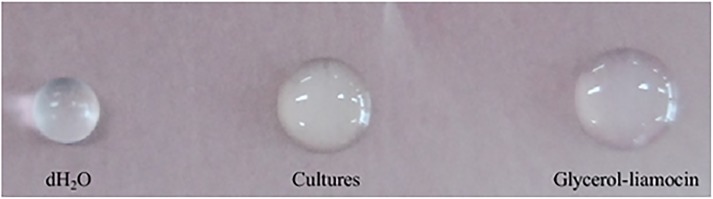
Picture illustrating the surface tension of glycerol-liamocin in aqueous solution dropped on a hydrophobic film.

To examine surfactant properties of glycerol-liamocin, a sample of culture medium and the size of glycerol-liamocin were compared with water as the control by the drop-collapse test (Fig 5).

When a surfactant with hydrophobic and hydrophilic moieties is added to water, surface tension decreases. In contrast, as intermolecular interactions increase, surface tension increases. Glycerol-liamocin exhibited a low surface tension of 31.5 mN/m at 1.5 mg/L, compared to that of water (72.8 mN/m). Furthermore, these results reveal that glycerol-liamocin exhibits lower surface tension than other surfactants, despite its low concentration. Thus, glycerol-liamocin was confirmed as an effective biosurfactant, with greater surfactant activity than other known surfactants [40].

The yield of glycerol-liamocin was very low compared to liamocins (0.5 to 6 g liamocins/L) [25]. In this study, we isolated and examined the compound showing the most potent biosurfactant-specific activity from *A*. *pullulans* L3-GPY culture broth, but there are many other major components with less activity than glycerol-liamocin that are under investigation.

In conclusion, *A*. *pullulans*, a microorganism isolated from a tiger lily flower, exhibited low surface tension activity. The active compound, glycerol-liamocin, was purified from the culture filtrate, and its chemical identity was determined to be a novel compound, based on extensive MS and NMR structural analyses. Further, purified glycerol-liamocin exhibited potent biosurfactant activity. In future studies, additional research on biosurfactant production by *A*. *pullulans* will enhance further understanding of potential biotechnological applications.

## Supporting Information

S1 Fig Numerical data of ^1^H NMR spectrum of glycerol-liamocin. (TIFF)

S2 Fig Numerical data of ^13^C NMR spectrum of glycerol-liamocin. (TIFF)

S3 Fig Numerical data of DEPT spectrum of glycerol-liamocin. (TIFF)

S4 Fig Numerical data of ^1^H-^1^H COSY spectrum of glycerol-liamocin. (TIFF)

S5 Fig A partial structure of glycerol-liamocin confirmed by numerical data of ^1^H- ^1^H COSY spectrum of glycerol-liamocin and shown using bold lines. (TIFF)

S6 Fig Numerical data of TOCSY spectrum of glycerol-liamocin. (TIFF)

S7 Fig A partial structure of glycerol-liamocin confirmed by numerical data of TOCSY spectrum of glycerol-liamocin and shown using bold lines. (TIFF)

S8 Fig Numerical data of HMQC spectrum of glycerol-liamocin. (TIFF)

S9 Fig Numerical data of HMBC spectrum of glycerol-liamocin. (TIFF)

S10 Fig A three-dimensional structure of glycerol-liamocin and numerical data of ^1^H NMR spectrum and ^13^C NMR spectrum. (TIFF)
