# Supplementary figures and images for: A Novel Biosurfactant Produced by Aureobasidium pullulans L3-GPY from a Tiger Lily Wild Flower, Lilium lancifolium Thunb
Source: PLoS One. 2015 Apr 7;10(4):e0122917. doi: 10.1371/journal.pone.0122917 (PMC4388725; doi:10.1371/journal.pone.0122917)

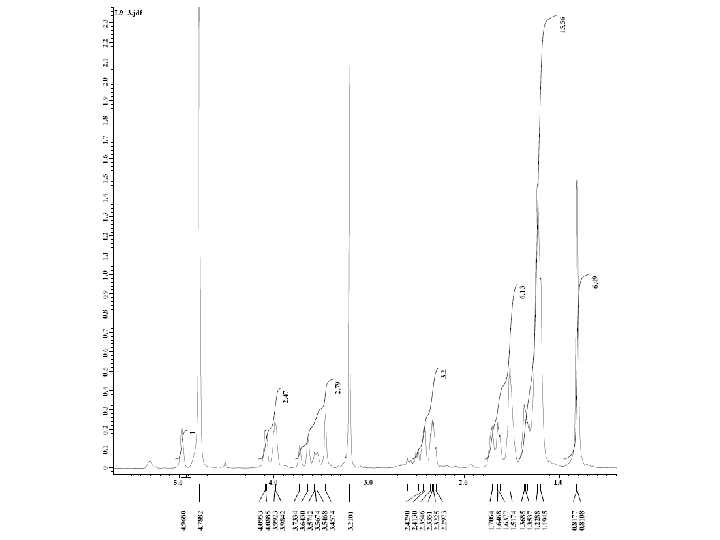

Supplement: S1 Fig — (TIFF) [file pone.0122917.s001.tiff]

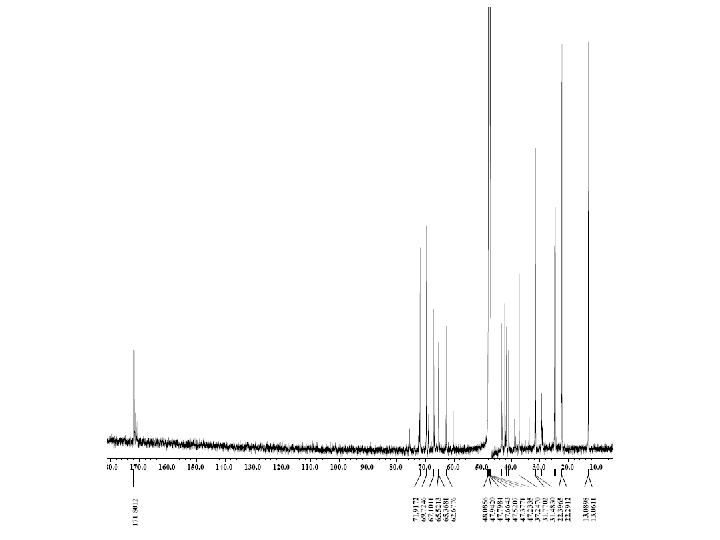

Supplement: S2 Fig — (TIFF) [file pone.0122917.s002.tiff]

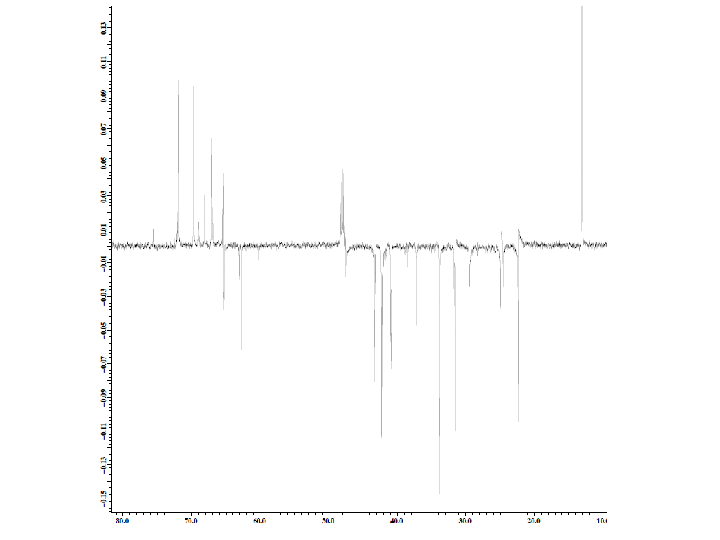

Supplement: S3 Fig — (TIFF) [file pone.0122917.s003.tiff]

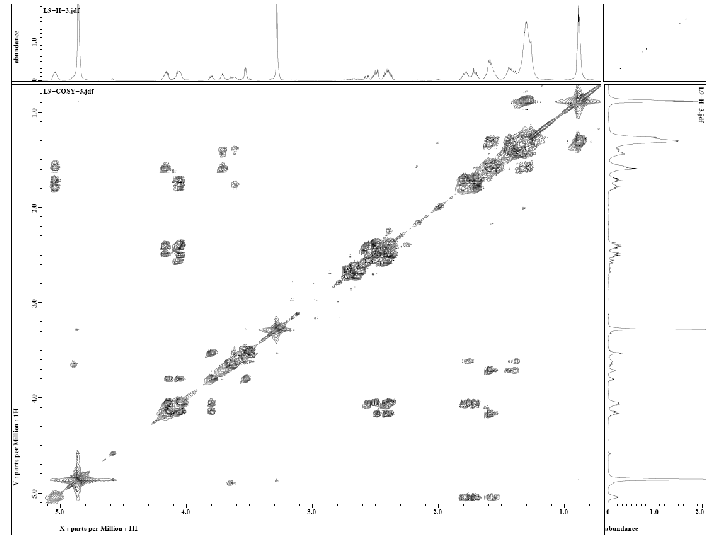

Supplement: S4 Fig — (TIFF) [file pone.0122917.s004.tiff]

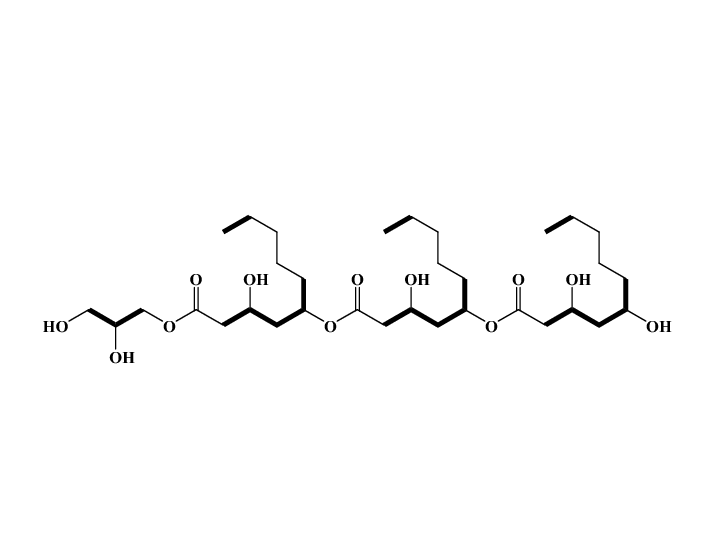

Supplement: S5 Fig — (TIFF) [file pone.0122917.s005.tiff]

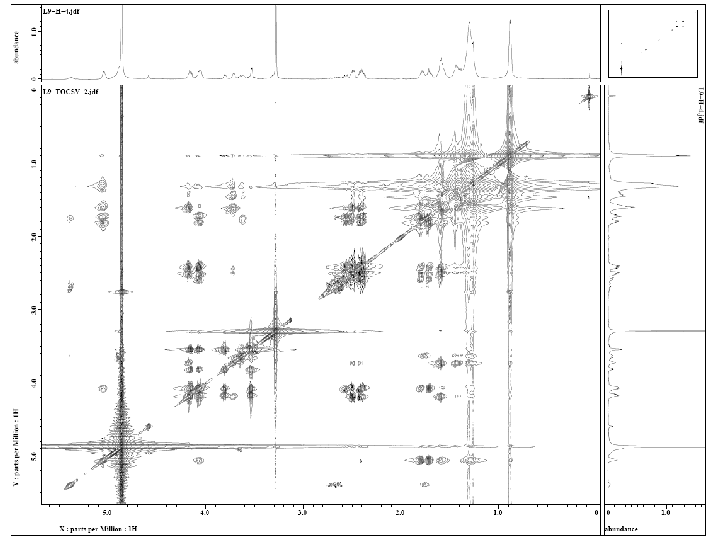

Supplement: S6 Fig — (TIFF) [file pone.0122917.s006.tiff]

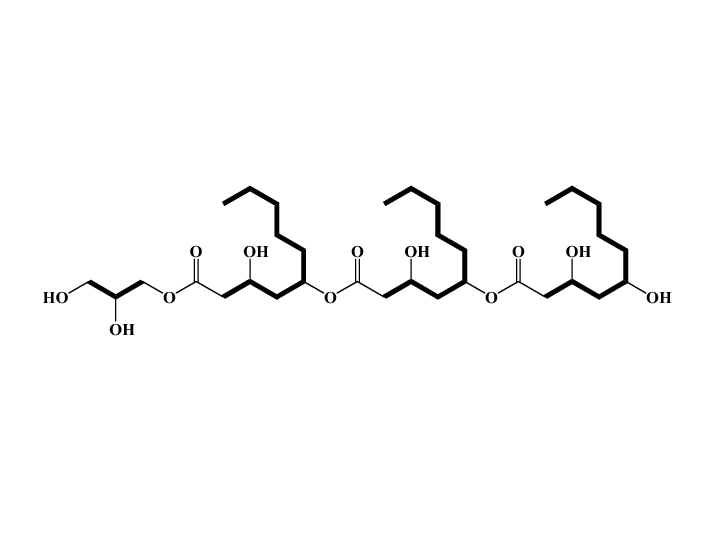

Supplement: S7 Fig — (TIFF) [file pone.0122917.s007.tiff]

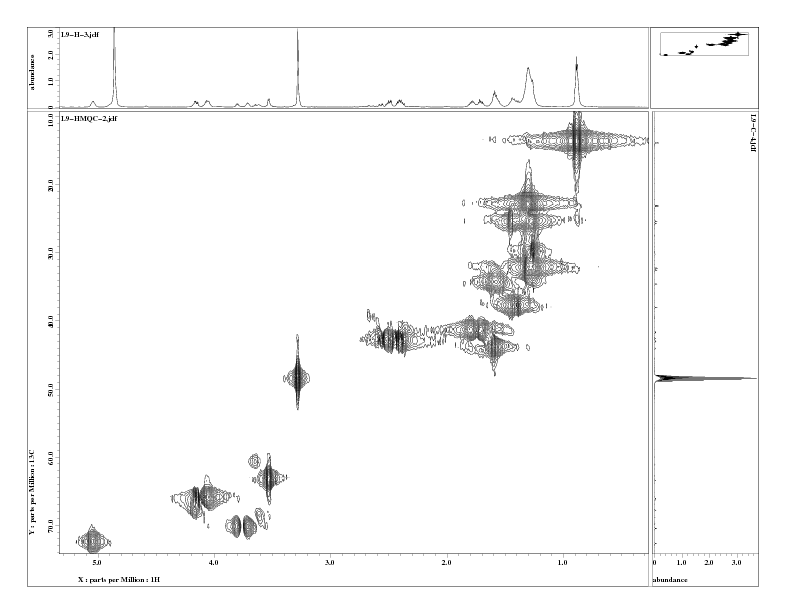

Supplement: S8 Fig — (TIFF) [file pone.0122917.s008.tiff]

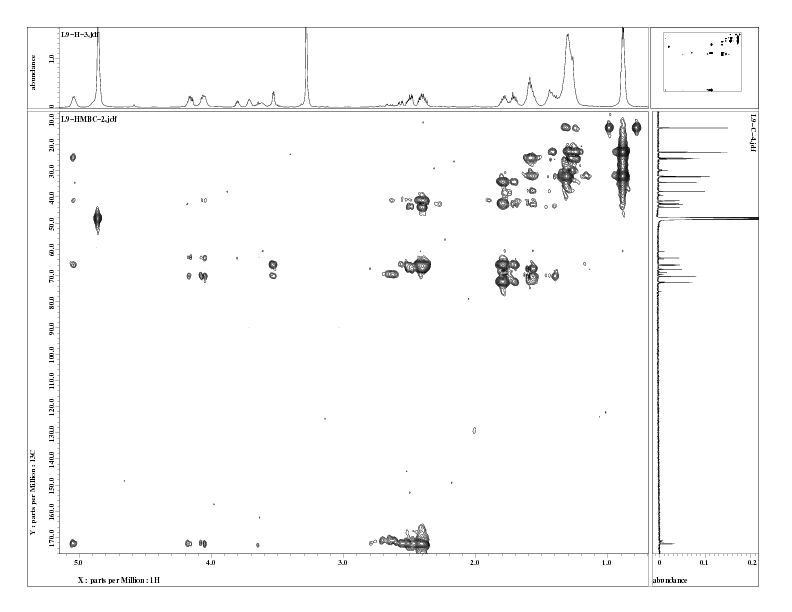

Supplement: S9 Fig — (TIFF) [file pone.0122917.s009.tiff]

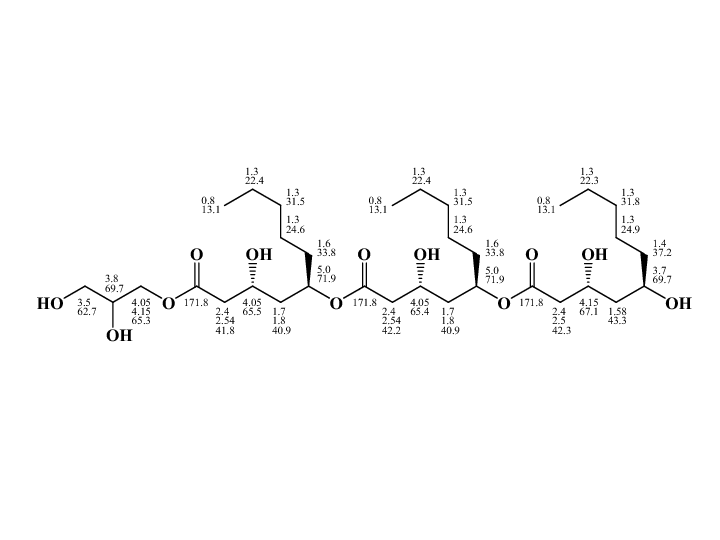

Supplement: S10 Fig — (TIFF) [file pone.0122917.s010.tiff]
